# The Relationship between Glycemic Index and Health

**DOI:** 10.3390/nu12020536

**Published:** 2020-02-19

**Authors:** Jennie Brand-Miller, Anette E. Buyken

**Affiliations:** 1 School of Life and Environmental Sciences and Charles Perkins Centre, University of Sydney, Sydney 2006, Australia; 2 Institute of Nutrition, Consumption and Health, Faculty of Natural Sciences, Paderborn University, 33098 Paderborn, Germany; anette.buyken@uni-paderborn.de

## 1. Introduction

There is no question that elevated postprandial glycemia is a significant driver of common chronic diseases globally. Studies such as DECODE [1] have linked impaired glucose tolerance to increased mortality, particularly from cardiovascular disease. The major cause of death in people with type 1 and type 2 diabetes is still cardiovascular disease. 

We also know that glucose tolerance can be improved by losing weight in persons with overweight or obesity, increasing physical activity and lean mass, and improving sleep quality. There are also nonmodifiable factors such as age, ethnicity, and family history that are associated with large differences in glucose tolerance and therefore postprandial glycemia (PPG). Despite these facts, there is still much confusion on how best to reduce PPG on a day-to-day basis by dietary means. 

In a single individual on a given day, carbohydrate-rich foods are the major determinant of PPG. A low-fat, high-carbohydrate meal will inevitably increase PPG more than a low-carbohydrate, high-protein and/or high-fat meal. Although this raises questions about the optimal carbohydrate and macronutrient energy distribution in our diet, the source of carbohydrate also plays a major role: gram-for-gram of carbohydrate, foods vary in their glycemic impact across a 10-fold range, from agave syrup with a glycemic index (GI) of 11 to Thai jasmin rice with a GI of around 100 (i.e., as large as the response to pure glucose, which serves as the comparator (=100)). 

Unfortunately, in 2020, 4 decades after the publication of the first comprehensive list of GI values, many health professionals still believe that sugar and sugar-sweetened beverages have greater impact on PPG than starchy foods like bread and potatoes. This belief has no scientific basis. Most starchy foods have a GI greater than 70, while most sugary foods are less than 70 [2], and on the whole, we eat twice as much energy in the form of starch as added sugar [3]. 

Yet, the relevance of the GI to health continues to be debated. One reason is that some consider that the GI is too variable between people, or that each individual has a unique physiology that means the average ranking of high to low GI foods is not applicable [4]. After 3 decades of testing the GI of hundreds of foods on a daily basis in thousands of individuals, we believe there is no such thing as an ‘individual GI’. Day-to-day variability in glucose tolerance is a more likely explanation for unexpected differences in glycemia. 

For this Special Issue of *Nutrients*, we encouraged the submission of original research or systematic reviews addressing the relationship between the GI and health outcomes. Twelve papers were forthcoming, each making a definitive contribution to new knowledge. 

In nutrition epidemiology, Livesey and members of the International Carbohydrate Quality Consortium undertook a dose response meta-analysis of prospective cohort studies on the relationship between GI and glycemic load (GL) and risk of type 2 diabetes [5]. They found that GI was robustly associated with incident type 2 diabetes, with the risk increasing by almost 90% when comparing the lowest to the highest exposure worldwide, i.e., overall dietary GI of 48 vs 76. This relative risk increase is much larger than the risk associated with consumption of too little fiber or too few wholegrains. 

In their second paper, Livesey et al. [6] consider the cause–effect relationship using the Bradford Hill criteria. They conclude that there is substantial evidence that GI and GL are causally linked to the risk of type 2 diabetes and that neither dietary fiber nor cereal fiber or wholegrains are reliable surrogate measures of GI or GL.

High GI diets have been related to increased risk of selected cancers in some studies but not others. Turati and colleagues [7] from Milan updated a previous meta-analysis, now including a total of 88 studies. High GI diets were associated with a summary RR of 1.2 for colorectal cancer and 1.25 for bladder cancer. Stomach, prostate and lung cancers were not associated with GI or GL. 

Edith Feskens and members of the PREVIEW Study Consortium developed a short food frequency questionnaire based on only 58 foods, which produced similar results for carbohydrate amount and GI as a much longer 183-item questionnaire [8] and is thus an attractive tool for assessing dietary GI in future epidemiological studies. 

The food industry has a role to play in bringing more healthy low GI foods to the table. One way is to add healthy ingredients that reduce glycemia. Interestingly, addition of a purified extract of figs was able to suppress glycemic and insulin responses to a standard meal [9]. The active component abscisic acid is found naturally in figs and other plants. 

The food industry also needs fast throughput in vitro methods that can roughly estimate GI during product development. Alexiandra et al. [10] used an in vitro methodology to simulate oral, gastric, and intestinal digestion and thereby predict the GI. Using this method, they found that simple methodological differences (e.g., use of glass balls) produced large differences in rate of digestion and therefore predicted GI. More research is urgently needed in this area. 

Reliable GI values can only be generated when standardised in vivo methodology is used. Thus, Wolever et al. compared the operation of the ISO standard for GI methodology in three different labs around the world. Three foods across a range of GI values were compared. There were no differences between labs, but the between-lab standard deviation was different for each food, ranging from 2 to 7. The findings indicate that the ISO method is sufficiently precise to distinguish foods that have a low GI (55 or less) from those with a high GI (70 or more) with 97%–99% probability.

Taken together, the contributions of this Special Issue illustrate that GI warrants consideration as a highly relevant measure of carbohydrate quality, along with the proportions of fiber, whole grain content, type of starch (resistant or not), and added vs. naturally-occurring sugars. A combined appraisal of all these dimensions is crucial when addressing the health relevance of carbohydrate quality.

We thank all of the contributors to this Special Issue of *Nutrients* and recommend their papers to you. 
